# Localization of quantitative trait loci for cucumber fruit shape by a population of chromosome segment substitution lines

**DOI:** 10.1038/s41598-020-68312-8

**Published:** 2020-07-03

**Authors:** Xiangfei Wang, Hao Li, Zhihui Gao, Lina Wang, Zhonghai Ren

**Affiliations:** State Key Laboratory of Crop Biology; Shandong Collaborative Innovation Center of Fruit & Vegetable Quality and Efficient Production; Key Laboratory of Biology and Genetic Improvement of Horticultural Crops in Huang-Huai Region, Ministry of Agriculture; College of Horticulture Science and Engineering, Shandong Agricultural University, Tai’an, 271018 Shandong People’s Republic of China

**Keywords:** Developmental biology, Genetics

## Abstract

Cucumber fruit shape, a significant agronomic trait, is controlled by quantitative trait loci (QTLs). Feasibility of chromosome segment substitution lines (CSSLs) is well demonstrated to map QTLs, especially the minor-effect ones. To detect and identify QTLs with CSSLs can provide new insights into the underlying mechanisms regarding cucumber fruit shape. In the present study, 71 CSSLs were built from a population of backcross progeny (BC_4_F_2_) by using RNS7 (a round-fruit cucumber) as the recurrent parent and CNS21 (a long-stick-fruit cucumber) as the donor parent in order to globally detect QTLs for cucumber fruit shape. With the aid of 114 InDel markers covering the whole cucumber genome, 21 QTLs were detected for fruit shape-related traits including ovary length, ovary diameter, ovary shape index, immature fruit length, immature fruit diameter, immature fruit shape index, mature fruit length, mature fruit diameter and mature fruit shape index, and 4 QTLs for other traits including fruit ground and flesh color, and seed size were detected as well. Together our results provide important resources for the subsequent theoretical and applied researches on cucumber fruit shape and other traits.

## Introduction

Cucumber (*Cucumis sativus* L., 2n = 14) is a worldwide important horticultural crop and has served as the research model plant of sex determination of Cucurbitaceae^1^. Cucumber fruit is a type of fleshy fruit that is usually consumed at immature stage (1–2 weeks after anthesis). In horticultural industry, fruit size and shape are important traits for selling and the determination of its usages^2^. According to the recently published papers, cucumber can be divided into six market classes or four geographic groups that exhibited extensive variations in shape or size: European long type, European short type, North/South China long type, North American short type, mini cucumber, and Japanese long type^2,3^.

Fruit size and shape are usually evaluated by the length (L) and diameter (D) of fruit, or its ratio (L/D), and are commonly modulated by both quantitative trait loci (QTLs) and some environmental factors^2,4,5^. Traditional QTL mapping, a reliable method of determining complex traits, was performed on fruit size and shape by analyzing F_2_, F_3_, BC (backcross) populations and recombinant inbred lines (RILs), and a series of expected QTLs were successfully detected^6–9^. For example, the first mapping for cucumber fruit shape-related QTLs was carried out by Kennard and Havey^10^ via using F_3_ and backcross populations, and 12 QTLs were identified to be involved in regulation of fruit length (FL), fruit diameter (FD) and the ratio of length to diameter (L/D), respectively. By using F_2_ and F_3_ populations, Serquen et al.^11^ detected 11 QTLs that were related with FL, FD, L/D ratio and fruit weight (FW). Recently, Bo et al.^12^ mapped 11 QTLs responsible for the regulation of FL, FD and FW using RIL populations developed from cultivated × semi-wild Xishuangbanna cucumber lines. Using three QTL models, Weng et al.^2^ mapped 12 consensus fruit size related QTLs with F_2_, F_3_ and RIL populations came from Gy14 (North American short fruit cucumber) × 9930 (North China long fruit cucumber) at multiple developmental stages and environments. By using two segregating populations from WI7200 (cultivated cucumber) × WI7167 (semi-wild Xishuangbanna cucumber), Pan et al.^13^ detected 21 QTLs that were involved in the regulation of mature fruit length (MFL), mature fruit diameter (MFD), FW and L/D ratio. With the populations originated from WI7238 (long fruit) × WI7239 (round fruit), Pan et al.^14^ detected two QTLs, *FS1.2* and *FS2.1*, which interacted with each other and exerted major effects on fruit shape. Further analysis revealed that *CsSUN* might be the candidate gene for *FS1.2*. Additionally, 10 possible candidate genes were identified in the *FS2.1* locus such as *CsTRM5*, which was an ortholog of *TRM5* gene in *Arabidopsis* and tomato^15^. More recently, three genes have been functionally validated. *CsFUL1*, a functional allele *FRUITFULL-like* MADS-box gene, was identified by analyzing the re-sequenced data of 150 cucumber lines, which regulates cucumber fruit length via exerting negative effects on the expression of *CsSUP* and auxin transporters^16^. Using two mutants from ethyl methanesulfonate mutagenesis, two fruit length controlling genes, *Short Fruit 1* (*SF1*) and *SF2* were isolated and functionally identified as a cucurbit-specific RING-type E3 ligase and a Histone Deacetylase Complex 1 (HDC1) homologue, respectively^17,18^.

To date, although five QTL/genes have already been cloned^14–18^, more QTL/genes need to be isolated and functionally validated^2,4,19^. Moreover, the molecular regulatory mechanism of cucumber fruit shape remains poorly understood. Conventional mapping populations such as F_2_, F_3_ and BC_1_, which are temporary ones, or RIL, which is permanent one, are usually used to identify QTLs with large effects, whereas minor effect or epistatic QTLs might be masked^20–22^. Therefore, the improvement of mapping populations has attracted continuously increased attentions from horticultural scientists and breeders^22–24^. Chromosome segment substitution line (CSSL), a kind of genetic material that randomly harbor a specific chromosomal segment of donor parent under the recipient genetic background, has been widely applied in a series of crop genetic research such as identification and mapping of QTLs associated with traits of interest^20,25^. Comparing with F_2_, F_3_, BC_1_ and RIL, CSSLs display a significant advantage that detection capacity of QTLs can be enhanced due to the elimination of blurring effects from multiple or interacting ones^26^. So far, lot of QTLs/genes of interest have been fine-mapped by using CSSLs in many plants such as maize^27–29^, cotton^30–32^, soybean^33–35^, *Brassica rapa*^22,36^, peanut^37^, wheat^38^, tomato^39–41^, and rice^42–45^.

In current study, a population consisting of 71 CSSLs was successfully built by using backcross progenies that came from a cross of CNS21 (long-stick-fruit) as the donor parent and RNS7 (round fruit) as the recurrent parent. CNS21, the Northern-China type inbred line has long stick commercial fruit (L/D > 10) with the average length of 36.20 ± 3.25 cm, green peel as well as white spines, while RNS7 sets round commercial fruit (L/D ≈ 1) with the average length of 7.30 ± 0.40 cm, white peel and black spines (Supplementary Table S3)^19^. A total of 114 InDel (insertion/deletions of DNA sequences) markers that showed polymorphisms between CNS21 and RNS7 were adopted in subsequent marker-assisted selection in order to identify fruit shape related QTLs. Totally 21 QTLs associated with fruit shape and 4 QTLs associated with fruit ground and flesh color, and seed size were detected. The results will facilitate the future fine mapping and cloning of these fruit related genes, thus benefiting our understanding about the genetic base of cucumber fruit related traits.

## Results

### Construction of cucumber CSSLs

**Figure 1:**
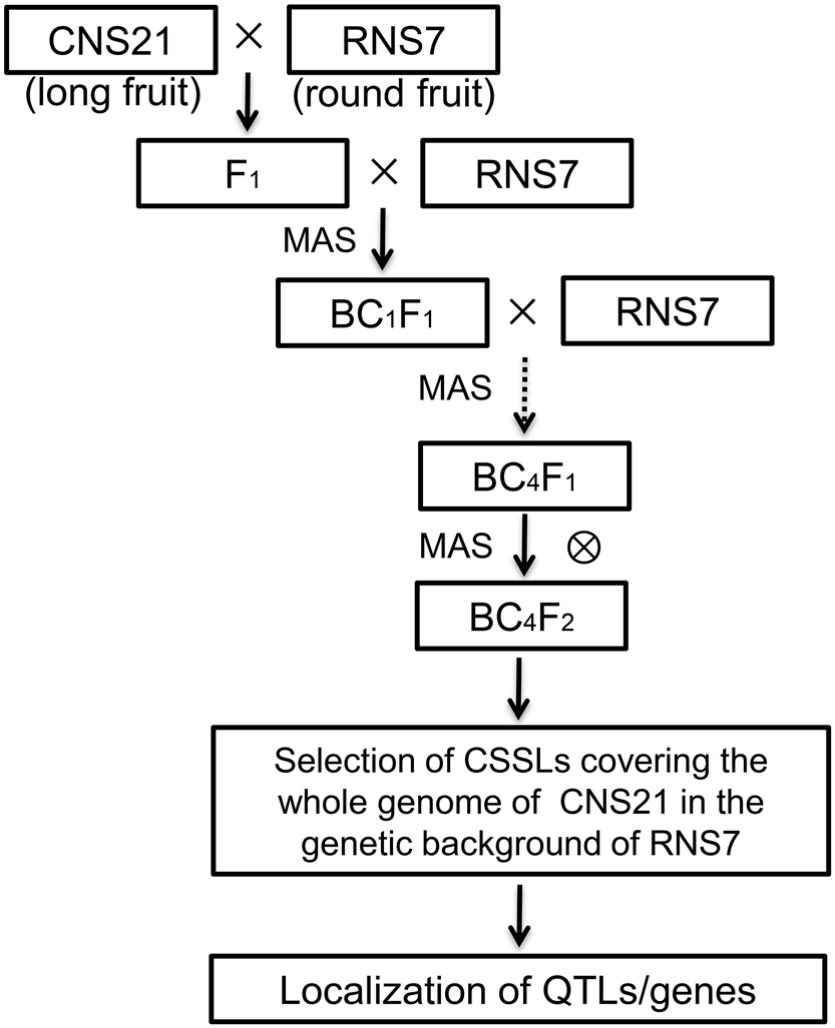
Schematic illustration for the construction of chromosome segment substitution lines (CSSLs) covering the whole cucumber genome. *QTLs* quantitative trait loci, *MAS* marker-assisted selection.

**Figure 2:**
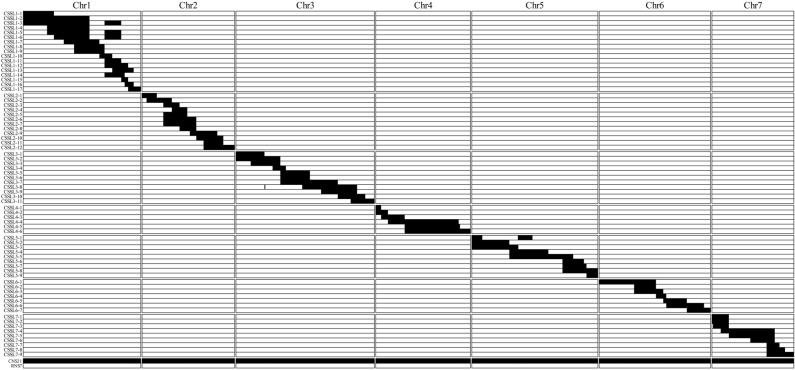
Schematic illustration for the genotypes of 71 CSSLs based on cucumber 9930 V3.0 draft genome. The white regions represent homologous segments from the recurrent parent, RNS7, and the black regions represent homologous segments from the donor parent, CNS21. CSSLs are indicated on the vertical axis.

The outline for cucumber CSSL construction was schematically illustrated in Fig. 1. To construct cucumber CSSL with RNS7 genetic background, the F_1_ plants from RNS7 (recurrent parent) × CNS21 (donor parent) were consecutively backcrossed to RNS7 four times in order to yield the BC_4_F_1_ generation. As a result, 500 BC_4_F_1_ individual plants were successfully obtained from 34 BC_3_F_1_ lines. All generations from BC_1_F_1_ to BC_4_F_1_ were screened using 114 InDel markers (Supplementary Fig. S1; Supplementary Table S4) based on the following criteria: (I) the most of genomes displayed high-level homozygosity with RNS7, except one to two substitutions from CNS21; (II) the selected individuals harbored less CNS21-derived chromosomal segments, which were able to cover the whole genome of CNS21 with overlapping regions between different ones. To obtain the desired CSSLs, 60 BC_4_F_1_ lines were self-pollinated and the resulted 1980 BC_4_F_2_ plants were further investigated by marker-assisted selection (MAS) based on cucumber 9930 V3.0 draft genome. A total of 71 independent BC_4_F_2_ substitution lines were kept as the cucumber CSSL population (Fig. 2).

### Characterization of substituted chromosomal segments in the CSSLs

**Figure 3:**
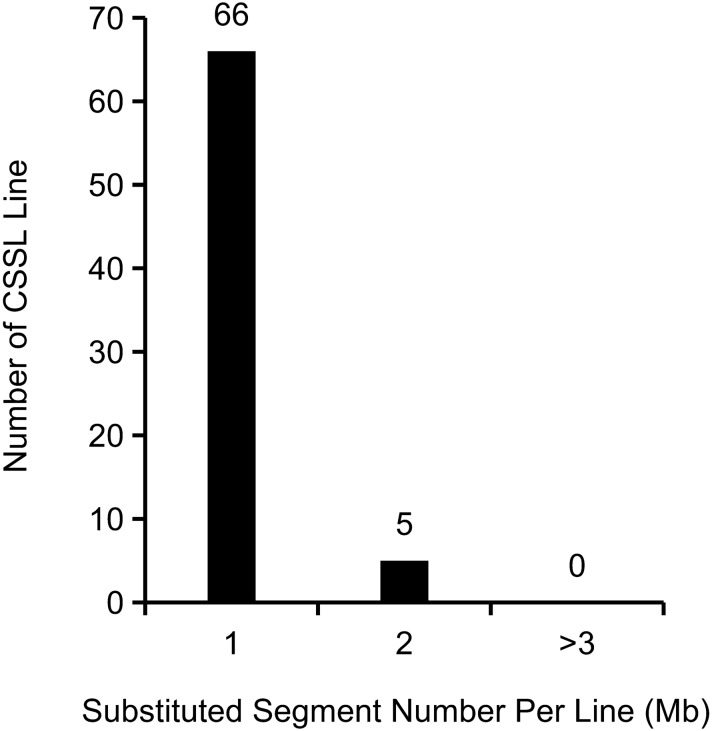
Occurrence frequency of substitution events in CSSLs.

**Figure 4:**
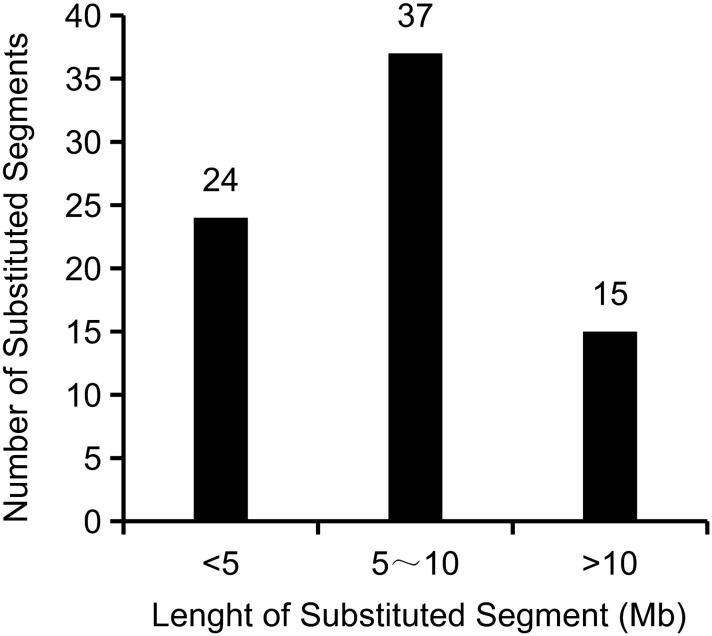
Size distribution of substituted chromosomal segments in CSSLs.

**Figure 5:**
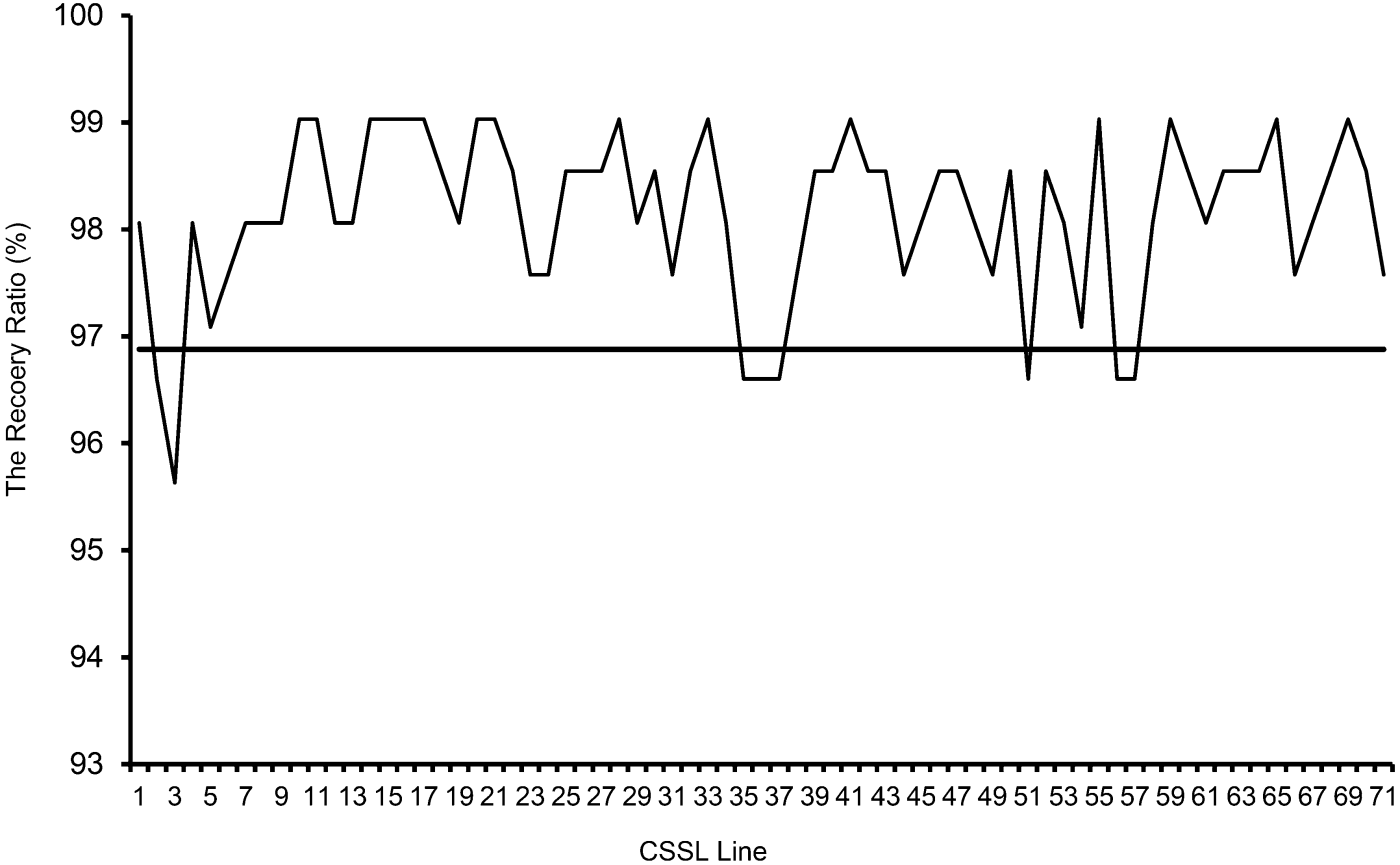
The recovery ratio of recurrent genome in each CSSL.

The 71 CSSLs, which possessed the genetic background of RNS7, totally harbored 76 substituted segments from CNS21. Thus this CSSL population contained 1.07 segments per line and 10.86 segments per chromosome on average (Fig. 3; Supplementary Table S1). Among these 71 CSSLs, 66 lines harbored only one CNS21-derived chromosomal segment, 5 harbored two segments, and none harbored three or more segments (Fig. 3). Numbers of the substituted segments were 20, 12, 12, 6, 10, 7 and 9 in chromosome 1 to chromosome 7, respectively (Supplementary Table S1). The summary length of CNS21-derived chromosomal segments in the CSSL population was approximately 546.96 Mb, which equated to 2.61 times of the sequenced genome size of cucumber (Supplementary Table S1). The length of substitutions in each chromosome ranged from 47.66 Mb (1.78 times of the chromosome size) in chromosome 4 to 140.81 Mb (4.28 times of the chromosome size) in chromosome 1, and was averaged as about 78.14 Mb (Supplementary Table S1). In each substitution line, the CNS21-derived chromosomal segments ranged in length from 1.73 to 19.31 Mb, with an average of 7.19 Mb (Supplementary Table S1). Among these CNS21-derived substitutions, 24 segments were smaller than 5 Mb, 37 were 5–10 Mb, and 15 were over 10 Mb (Fig. 4). The recovery ratio of the 71 CSSLs ranged from 95.63 to 99.03% (Fig. 5).

### Identification of QTLs for fruit shape

**Figure 6:**
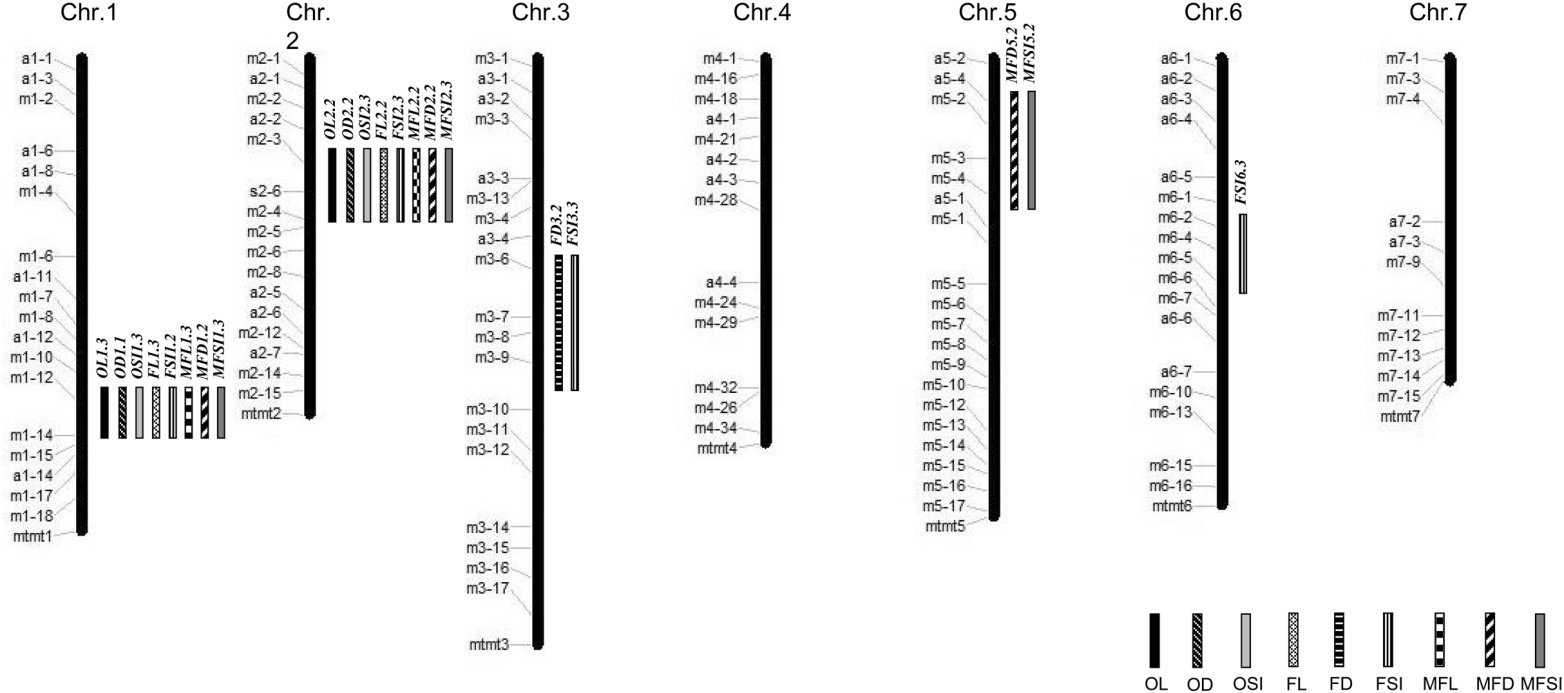
Chromosomal distribution of 21 QTLs for cucumber fruit shape. DNA markers and their physical locations are indicated on the left side of each chromosome based on cucumber 9930 V3.0 draft genome. Short stripes filled with different hatched regions on right sides of chromosomes represent the locations of different QTLs.

**Figure 7:**
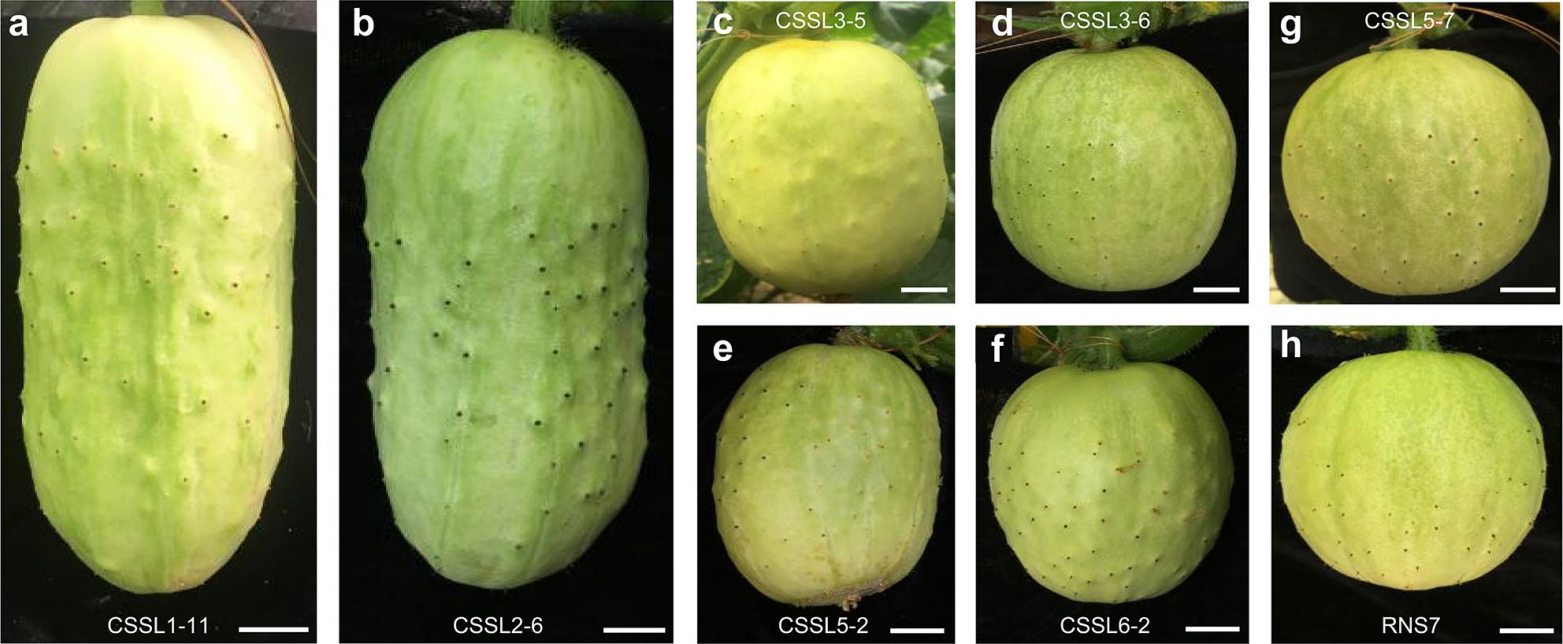
Fruit phenotypes of CSSLs carrying QTLs for fruit shape. (**a**–**f**) CSSLs carrying QTLs for fruit shape. (**g**) A CSSL not carrying QTLs for fruit shape. (**h**) The recurrent parent RNS7. Scale bar = 1 cm.

To identify the chromosomal segments involved in fruit shape, phenotypic variations of fruit shape related parameters including fruit length, fruit diameter and the ratio of length to diameter were investigated in the CSSL population at anthesis, commercial and mature fruit stages (Supplementary Table S3). The fruit shape was markedly different between the two parents, and the L/D index of CNS21 was consistently greater than that of RNS7^19^. In the CSSL population, the fruit length and fruit diameter segregated significantly at immature fruit stage, ranging from 44 to 111 mm and 36.5 to 80 mm, respectively (Table 1; Supplementary Table S3; Fig. 7). The similar results were found at anthesis and mature fruit stages as well (Table 1; Supplementary Table S3). Of these CSSLs, fruit shape of 10 lines were dramatically different from RNS7 (Table 1). By analyzing the CSSL population, we detected 21 fruit shape related QTLs on chromosomes 1, 2, 3, 5 and 6, which included 2 responsible for ovary length (OL), 2 for ovary diameter (OD), 2 for ovary shape index (OSI), 2 for commercial fruit length (FL), 1 for commercial fruit diameter (FD), 4 for commercial fruit shape index (FSI), 2 for mature fruit length (MFL), 3 for mature fruit diameter (MFD) and 3 for mature fruit shape index (FSI) (Fig. 6). Of these 21 QTLs, 16 were detected in the region of 22.73–28.27 Mb on chromosome 1 and the region of 5.10–14.23 Mb on chromosome 2, respectively (Fig. 6; Supplementary Fig. S4; Table 1). QTLs for OL, OD, OSI, FL, FSI, MFL, MFD and MFSI were identified in the aforementioned regions (Fig. 6; Table 1), confirming the great contributions of loci on chromosomes 1 and 2 to fruit shape (Supplementary Fig. S4). In addition, one QTL for FD was mapped in the region of 16.22–22.78 Mb on chromosome 3 as well (Fig. 6; Table 1). Two QTLs for FSI were mapped in the region of 16.22–22.78 Mb on chromosome 3 and 11.70–17.62 Mb on chromosome 6 (Fig. 6; Table 1). Two QTLs for MFD and MFSI were detected in the region of 0–10.61 Mb on chromosome 5 (Fig. 6; Table 1).

**Table 1:** Phenotypic comparisons and additive effects of CSSLs carrying QTLs for fruit shape.

Line	Chr	Position (Mb)^a^	Substituted region	Trait (mm)	% variation (R^2^)	Add
**OL**						
CSSL1-11	1	22.73–23.00	m1-12 to m1-14	21.30 ± 0.58***	55.92	5.15
CSSL1-12	1	22.73–28.27	m1-12 to m1-15	17.25 ± 2.99*	55.92	3.13
CSSL2-4	2	8.24–11.43	s2-6 to m2-4	18.00 ± 1.00**	35.75	3.50
CSSL2-5	2	5.10–11.43	m2-3 to m2-4	19.25 ± 0.96***	35.75	4.13
CSSL2-7	2	5.10–14.23	m2-3 to m2-6	18.33 ± 0.58***	35.75	3.67
CSSL2-8	2	10.18–14.23	m2-4 to m2-6	16.33 ± 1.50**	35.75	2.67
RNS7				11.00 ± 0.50		
**OD**						
CSSL1-11	1	22.73–23.00	m1-12 to m1-14	7.20 ± 0.29***	31.58	− 1.90
CSSL1-12	1	22.73–28.27	m1-12 to m1-15	7.80 ± 0.96*	31.58	− 1.60
CSSL2-4	2	8.24–11.43	s2-6 to m2-4	7.70 ± 0.58**	24.00	− 1.65
CSSL2-5	2	5.10–11.43	m2-3 to m2-4	7.50 ± 0.58**	24.00	− 1.75
CSSL2-7	2	5.10–14.23	m2-3 to m2-6	7.20 ± 0.29***	24.00	− 1.90
CSSL2-8	2	10.18–14.23	m2-4 to m2-6	7.30 ± 0.58**	24.00	− 1.85
RNS7				11.00 ± 1.00		
**OSI**						
CSSL1-11	1	22.73–23.00	m1-12 to m1-14	2.98 ± 0.17***	52.33	0.97
CSSL1-12	1	22.73–28.27	m1-12 to m1-15	2.24 ± 0.42*	52.33	0.60
CSSL2-4	2	8.24–11.43	s2-6 to m2-4	2.36 ± 0.22**	43.67	0.66
CSSL2-5	2	5.10–11.43	m2-3 to m2-4	2.57 ± 0.10***	43.67	0.76
CSSL2-7	2	5.10–14.23	m2-3 to m2-6	2.56 ± 0.02***	43.67	0.76
CSSL2-8	2	10.18–14.23	m2-4 to m2-6	2.23 ± 0.17**	43.67	0.59
RNS7				1.05 ± 0.03		
**FL**						
CSSL1-11	1	22.73–23.00	m1-12 to m1-14	96.00 ± 9.00**	40.71	11.50
CSSL1-12	1	22.73–28.27	m1-12 to m1-15	88.00 ± 7.00**	40.71	7.50
CSSL2-4	2	8.24–11.43	s2-6 to m2-4	103.00 ± 13.00*	44.61	15.00
CSSL2-5	2	5.10–11.43	m2-3 to m2-4	100.00 ± 7.00**	44.61	13.50
CSSL2-7	2	5.10–14.23	m2-3 to m2-6	111.00 ± 8.00**	44.61	19.00
CSSL2-8	2	10.18–14.23	m2-4 to m2-6	93.00 ± 11.00*	44.61	10.00
RNS7				73.00 ± 4.00		
**FD**						
CSSL3-6	3	16.22–22.78	m3-6 to m3-9	36.50 ± 0.71**	6.70	− 20.00
RNS7				76.50 ± 3.50		
**FSI**						
CSSL1-11	1	22.73–23.00	m1-12 to m1-14	2.05 ± 0.05***	38.25	0.55
CSSL1-12	1	22.73–28.27	m1-12 to m1-15	1.84 ± 0.02***	38.25	0.45
CSSL2-4	2	8.24–11.43	s2-6 to m2-4	2.64 ± 0.23**	53.22	0.85
CSSL2-5	2	5.10–11.43	m2-3 to m2-4	1.81 ± 0.04***	53.22	0.43
CSSL2-7	2	5.10–14.23	m2-3 to m2-6	2.53 ± 0.13**	53.22	0.79
CSSL2-8	2	10.18–14.23	m2-4 to m2-6	2.12 ± 0.07**	53.22	0.59
CSSL3-6	3	16.22–22.78	m3-6 to m3-9	1.34 ± 0.03**	4.68	0.20
CSSL6-2	6	11.70–16.39	m6-2 to m6-5	1.30 ± 0.01**	8.86	0.18
CSSL6-3	6	11.70–17.62	m6-2 to m6-6	1.23 ± 0.01**	8.86	0.14
RNS7				0.95 ± 0.01		
**MFL**						
CSSL1-11	1	22.73–23.00	m1-12 to m1-14	128.00 ± 17.00*	40.42	18.50
CSSL1-12	1	22.73–28.27	m1-12 to m1-15	142.00 ± 24.00*	40.42	25.50
CSSL2-4	2	8.24–11.43	s2-6 to m2-4	171.00 ± 16.00**	39.94	40.00
CSSL2-5	2	5.10–11.43	m2-3 to m2-4	173.00 ± 23.00*	39.94	41.00
CSSL2-7	2	5.10–14.23	m2-3 to m2-6	124.00 ± 20.00*	39.94	16.50
CSSL2-8	2	10.18–14.23	m2-4 to m2-6	156.00 ± 16.00*	39.94	32.50
RNS7				91.00 ± 4.00		
**MFD**						
CSSL1-11	1	22.73–23.00	m1-12 to m1-14	64.00 ± 5.00**	19.70	− 19.75
CSSL1-12	1	22.73–28.27	m1-12 to m1-15	84.00 ± 2.00*	19.70	− 9.75
CSSL2-4	2	8.24–11.43	s2-6 to m2-4	76.00 ± 5.00*	22.39	− 13.75
CSSL2-5	2	5.10–11.43	m2-3 to m2-4	82.00 ± 9.00*	22.39	− 10.75
CSSL2-7	2	5.10–14.23	m2-3 to m2-6	65.00 ± 5.00**	22.39	− 19.25
CSSL2-8	2	10.18–14.23	m2-4 to m2-6	79.00 ± 9.00*	22.39	− 12.25
CSSL5-2	5	0.00–10.61	a5-4 to m5-4	85.00 ± 5.00*	9.65	− 9.25
RNS7				103.50 ± 8.50		
**MFSI**						
CSSL1-11	1	22.73–23.00	m1-12 to m1-14	1.99 ± 0.10**	49.23	0.56
CSSL1-12	1	22.73–28.27	m1-12 to m1-15	1.70 ± 0.24*	49.23	0.41
CSSL2-4	2	8.24–11.43	s2-6 to m2-4	2.26 ± 0.07***	55.68	0.69
CSSL2-5	2	5.10–11.43	m2-3 to m2-4	2.12 ± 0.04***	55.68	0.62
CSSL2-7	2	5.10–14.23	m2-3 to m2-6	1.94 ± 0.46*	55.68	0.53
CSSL2-8	2	10.18–14.23	m2-4 to m2-6	1.99 ± 0.04***	55.68	0.56
CSSL5-2	5	0.00–10.61	a5-4 to m5-4	1.09 ± 0.02**	9.65	0.11
RNS7				0.88 ± 0.04		

*Add* additive effect, *OL* ovary length, *OD* ovary diameter, *OSI* ovary shape index, *FL* fruit length (commercial stage), *FD* fruit diameter (commercial stage), *FSI* fruit shape index (commercial stage), *MFL* mature fruit length, *MFD* mature fruit diameter, *MFSI* mature fruit shape index.

*indicates significant differences at the statistical level of 0.05, **indicates significant differences at the statistical level of 0.01, ***indicate significant differences at the statistical level of 0.001. Traits of interest were described as the means ± standard deviations (n ≥ 5).

^a^Based on cucumber 9930 V3.0 draft genome.

### Identification of QTLs for seed shape and fruit color

In addition to fruit shape, the two parents showed significant differences in other fruit traits such as commercial fruit ground color (FGC), fruit flesh color (FLC) and seed shape^19^. Using CSSL2-7, two QTLs for seed length (SDL) and seed width (SW) were identified in the region of 5.10–14.23 Mb on chromosome 2 (Supplementary Fig. S2; Supplementary Table S2). Using CSSL3-11, two QTLs associated with FGC and FLC were uncovered in the 33.31–40.88 Mb region of chromosome 3 (Supplementary Fig. S3; Supplementary Table S2).

## Discussion

Fruit shape/size, an important quality trait in cucumber, is often affected by both genetic composition and environmental conditions. To date, there is little information available on the genetic mechanisms of fruit shape/size. CSSLs are ideal materials to detect QTLs and evaluate their contributions to the trait of interest as a single Mendelian factor. CSSLs have extensively been applied for the identification of genes that control important agronomic traits in rice^46,47^, maize^28,29^, *Brassica rapa*^22,36^, tomato^40,41^, and so on. However, thus far, only three sets of cucumber CSSLs have already been constructed. The first set of CSSLs was created through a cross of the wild cucumber PI183967 (donor) and the cultivated line Xintaimici (receptor), providing new resources for utilization of valuable genes from wild cucumber^48^. The other set was adopted to detect powdery mildew (PM) resistance-related genes^49^. The third set is in the present study (Fig. 2). Polymorphic marker density across whole genome profoundly influences the quality of CSSLs and thus plays crucial roles in the creation of CSSLs^50^. The CSSLs constructed by Li et al.^48^ only contain 31 lines including 10 lines harboring two substitution segments, and their substitution segments were big because of small number of CSSLs and makers used in selection. Although the CSSLs for detecting PM resistance-related genes have 17 families with 499 plants, only two markers, one is associated with dwarf plants and the other with PM resistance, were used in the construction of the CSSLs^49^. However, 114 InDel markers that were distributed on the 7 chromosomes relatively evenly were adopted for the construction of CSSLs in the current study (Supplementary Fig. S1). Moreover, 66 of the 71 CSSLs contained single substituted segment and the other 5 lines were identified to contain two substituted segments (Fig. 3). The length of substituted chromosomal segments in each line ranged from 1.73 to 19.31 Mb, and the average value of these segments was approximately 7.19 Mb (Supplementary Table S1). So these lines harbored a high recovery rate of 95.63–99.03% of the recurrent parent genome and simultaneously the genetic background noise was tremendously decreased (Fig. 5), thus being considered as a powerful tool to identify, map and validate QTLs of interest.

Using the CSSL population, totally, 21 QTLs responsible for cucumber fruit shape were identified and of which, eight QTLs were detected in the region of 22.73–28.27 Mb on chromosome 1 (Fig. 6; Table 1), where numerous QTLs for OL, OD, OSI, FL, FSI, MFL, MFD and MFSI were detected in previous studies (Fig. 6; Supplementary Fig. S4)^19^. As the best candidate of *FS1.2*, *CsSUN* was located in this region, being a major QTL of fruit shape (Fig. 7a)^14,19^. Comparing with previously reported regions on chromosome 1^19,51^, the size of estimated QTL region in our research was much smaller (Supplementary Fig. S4). The QTLs for OL, OD, OSI, FL, FSI, MFL, MFD and MFSI were identified at the long arm of chromosome 2 in the present study (Fig. 6; Supplementary Fig. S4). The chromosome 2 region harboring these detected QTLs displayed overlapping, but much smaller than the previous three reports by Weng et al.^2^, Gao et al.^19^ and Pan et al.^51^. This QTL(s) on chromosome 2 was (were) uncovered as (a) major one(s) by Pan et al.^14^, and our data provided a direct evidence for *FS2.1* being a major QTL (Fig. 7b; Table 1). Wu et al.^15^ reported that there were 10 possible candidate genes in *FS2.1* locus such as *CsTRM5*, an ortholog of tomato *TRM5* that was able to balance the *OVATE* and *SlOFP20*-mediated cell division patterns to determine the final tomato shapes. It was thus that *CsTRM5* was regarded as the most possible candidate gene for *FS2.1*, but they did not give direct genetic evidence of sequence difference or gene expression between the two parental lines used in their research^15^. Our resequencing results revealed that some single nucleotide polymorphisms (SNPs) or InDels in *CsSUN* and *CsTRM5* genes, which were just located on the region of 22.73–28.27 Mb on chromosome 1 and the region of 5.10–14.23 Mb on chromosome 2 respectively, between RNS7 and CNS21 (data not shown). We thus speculate that *CsSUN* and *CsTRM5* genes could be the candidate genes for QTLs on chromosomes 1 and 2, respectively. However, more experimental evidence should be provided in future study to support this assumption. In addition, *SF1* was localized in the region of 5.10–14.23 Mb on chromosome 2 in which *FS2.1* was mapped in our study and previous studies (Supplementary Fig. S4)^17,19^. However, there is no difference in protein sequence and gene expression of *SF1* between RNS7 and CNS21. The identification of major QTLs (*R*^2^ > 10%) on shortened regions of chromosomes 1 and 2 in the present study (Table 1; Supplementary Fig. S4) indicated that CSSLs could be an advantageous tool for fine mapping stable QTLs and give more information about these QTLs under different cucumber genetic backgrounds.

Furthermore, the CSSLs harboring a single segment substitution make it feasible to mine minor-effect QTLs^21,25,52–54^. In the current study, five minor-effect QTLs (*R*^2^ ≤ 10%) associated with fruit shape were detected on chromosomes 3, 5 and 6 (Figs. 6, 7c–f; Table 1) and displayed a relatively complex relationship with previous studies^2,12,13,19,51,55^. In most cases, the identified minor-effects regions on the three chromosomes were well consistent with those described previously^2,12,13,51,55^, while the inconsistency was also revealed for *FD3.2* and *FSI3.3* on chromosome 3 with the detected effect regions on the same chromosome by Wei et al.^55^ and Pan et al.^13^, possibly due to the differences in genetic background, traits of interest or environmental conditions (Fig. 6; Supplementary Fig. S4). Up to date, none of them has yet been fine mapped and cloned because that it is scarcely possible to fine map or clone these minor QTLs using the F_2_, F_3_, BC or RIL populations. However, the CSSLs that we constructed in this present study provided an opportunity for isolating the minor QTLs related to cucumber fruit shape.

We also detected QTLs that were associated with seed size and fruit color in the present study (Supplementary Table S2). The QTLs for SDL and SW were identified in the same region for OL, OD, OSI, FL, FSI, MFL, MFD and MFSI on chromosome 2, suggesting that *FS2.1* might have pleiotropic effects (Supplementary Fig. S2; Supplementary Fig. S4; Table 1; Supplementary Table S2). More recently, two consensus QTLs (*CsSS2.1* and *CsSS2.2*) associated with seed size have been reported on chromosome 2 in a review paper by Guo et al.^56^, and *CsSS2.1* displays overlapping, but larger than the identified QTLs for SDL and SW in the present study. The smaller QTL regions in this study will facilitate the future fine-mapping for genes responsible for seed size. The QTLs for FGC and FLC were observed in the distal region of chromosome 3 (Supplementary Fig. S3; Supplementary Table S2), being consistent with the previous results reported by Liu et al.^57,58^ and Tang et al.^59^. The *w* gene controlling white immature fruit color was localized in this region of chromosome 3, but no difference in coding sequence (CDS) of *w* was observed between RNS7 and CNS21. It will be very intriguing to reveal more candidate genes responsible for fruit color in future studies. In addition, QTLs related to other agronomic traits could be identified with these CSSLs.

In summary, we created a set of CSSLs that resulted from a cross between RNS7 (a round-fruit line) and CNS21 (a long-stick-fruit line) using 114 InDel markers covering the whole cucumber genome (9930 V3.0). Using these CSSLs, we identified 25 QTLs related to fruit shape, fruit color and seed size. Our study provides a powerful tool to isolate the QTLs for fruit shape, especially the minor ones, and other agronomic trait QTLs.

## Materials and methods

### Plant materials and growth conditions

Two parents CNS21 and RNS7, were used to construct the CSSL population^19^. Seeds of two parents were germinated in darkness at 28 °C overnight in petri dishes and grown in a growth chamber that was programed as photoperiod of 16 h, air temperature of 25 °C over light course and of 18 °C over dark course. Cucumber seedlings were transferred to a greenhouse of Shandong Agricultural University when they were grown to two-leaf stage. Standard field managements were carried out over cucumber cultivation course.

### Molecular marker development

A total of 114 InDel markers that were distributed evenly throughout the cucumber (Chinese Long) 9930 V3.0 genome (https://cucurbitgenomics.org/organism/20) were developed from the data of sequenced genomes (Supplementary Fig. S1)^19^. And 18, 16, 19, 14, 19, 17 and 11 InDel markers were located on chromosome 1 to chromosome 7, respectively (Supplementary Fig. S1). The average distance was approximately 1.85 Mb between two neighboring markers on the same chromosome. The primers used in the present study were listed in Supplementary Table S4.

### Construction of CSSLs

The schematic illustration for construction of CSSLs was displayed in Fig. 1. The F_1_ plants were generated from a cross between CNS21 and RNS7. Then consecutive backcross was performed between the F_1_ plants and RNS7 four times in order to generate the BC_4_F_1_. Over the course from BC_1_F_1_ to BC_4_F_1_, the genotype of each individual was analyzed by marker-assisted selection (MAS), and labelled as ‘B’ if the genotype was the same as ‘RNS7’, or as ‘H’ if the genotype was heterogeneous. The appropriate ‘H’ individuals in each generation were further chosen out based on the criteria of harboring CNS21-derived chromosomal segments as well as these segments covering whole cucumber genome, and finally 60 BC_4_F_1_ individuals were selected from 500 BC_4_F_1_ plants. Thereafter, BC_4_F_2_ population was generated by self-pollination of the selected BC_4_F_1_ plants for further MAS analysis based on the following principles: the substitution of chromosomal segment in RNS7 by a single CNS21-derived segment, the maintenance of genetic background at a high-level homozygosity with RNS7, and the existence of partially overlapping between substituted chromosomal segments. 114 InDel markers were applied in the method of MAS during the selection process. Ultimately, 71 BC_4_F_2_ lines were chosen from 1980 BC_4_F_2_ individuals to create a set of CSSLs for the further mapping of cucumber fruit trait QTLs.

### DNA extraction and genotype analysis

Genomic DNAs were extracted from unexpanded young leaves of each plant following the CATB protocol reported by Murray and Thompson^60^. Then the above-mentioned 114 InDel markers were applied to detect the individuals over foreground and background selections. The target DNA segments were amplified on a ABI PCR machine (Thermo Fisher Scientific, USA) with the correspond InDel markers. The resulted products were separated on a 3.5% (W/V) agarose gel and photographed with a FR-980A image analysis system (Shanghai Furi Science and Technology, China).

### Phenotypic analysis

Phenotypic data of CSSLs and RNS7 were recorded in the solar greenhouse of Shandong Agricultural University over three years (2016, 2017 and 2018). Two self-pollinated fruits were allowed on each plant. Fruit length (L), fruit diameter (D) and the ratio of length to diameter (L/D) were determined at three developmental stages: ovary length (OL), ovary diameter (OD) and ovary shape index (OSI) at anthesis; commercial fruit length (FL), fruit diameter (FD) and fruit shape index (FSI) at 10–12 days post pollination (dpp), and mature fruit length (MFL), mature fruit diameter (MFD) and mature fruit shape index (MFSI) at 45–55 dpp. At least 5 biological repeats were performed to collect all data. For each repeat, five to ten typical fruits at anthesis, three to five typical fruits at immature fruit stage, and two typical fruits at mature fruit stage, respectively, were selected for statistical analysis of phenotypic parameters including fruit length (L), fruit diameter (D) and the ratio of length to diameter. Seed length (SDL) and seed width (SW) were collected from at least 20 seeds. Data analyses were performed with statistical algorisms installed in MICROSOFT Excel 2013.

### QTL mapping

Given there were significant differences in the average value of a trait between a CSSL and RNS7, the existence of QTLs was further estimated. The detection of QTLs was performed on the basis of the *t*-test results that were derived from the difference comparison between the mean values of each CSSL and RNS7 (*P* value ≤ 0.05). The additive effect of individual QTL was evaluated by following the formula below^53^: Additive effect = 1/2 × (value of CSSL-value of RNS7).

The observed phenotypic variance (*R*^2^), a parameter commonly adopted to evaluate the effect strength of a given QTL, was calculated for these detected QTLs by using the QTL IciMapping V4.1 software with previously introduced settings^19^. The QTLs with over 10% of *R*^2^ were defined as major-effect ones and the others were defined as minor-effect ones according to the previous study^19^.

## Supplementary information

Supplementary file1

Supplementary file2

## Abbreviations

BC: Backcross
CDS: Coding sequence
CSSL: Chromosome segment substitution line
D: Diameter
dpp: Days post-pollination
FD: Fruit diameter
FGC: Fruit ground color
FL: Fruit length
FLC: Flesh color
FSI: Fruit shape index
FW: Fruit weight
InDel: Insertion/deletions of DNA sequence
L: Length
MAS: Marker-assisted selection
MFD: Mature fruit diameter
MFL: Mature fruit length
MFSI: Mature fruit shape index
OD: Ovary diameter
OL: Ovary length
OSI: Ovary shape index
PCR: Polymerase chain reaction
PM: Powdery mildew
QTL: Quantitative trait locus
RIL: Recombinant inbred line
SDL: Seed length
SNP: Single nucleotide polymorphism
SW: Seed width
