# Supplementary material for: Localization of quantitative trait loci for cucumber fruit shape by a population of chromosome segment substitution lines
Source: Sci Rep. 2020 Jul 3;10:11030. doi: 10.1038/s41598-020-68312-8 (PMC7334212; doi:10.1038/s41598-020-68312-8)
Supplement: Supplementary file 1 — Supplementary file1 [file 41598_2020_68312_MOESM1_ESM.docx]

**Localization of Quantitative Trait Loci for Cucumber Fruit Shape by A Population of Chromosome Segment Substitution Lines**

Xiangfei Wang^†^, Hao Li^†^, Zhihui Gao, Lina Wang*, Zhonghai Ren*

State Key Laboratory of Crop Biology; Shandong Collaborative Innovation Center of Fruit & Vegetable Quality and Efficient Production; Key Laboratory of Biology and Genetic Improvement of Horticultural Crops in Huang-Huai Region, Ministry of Agriculture; College of Horticulture Science and Engineering, Shandong Agricultural University, Tai’an, Shandong 271018, People’s Republic of China

†These authors contribute equally to this work.

*Correspondence should be addressed to Z. R. ([zhren@sdau.edu.cn](mailto:zhren@sdau.edu.cn)) or L.W. ([lnwang@sdau.edu.cn](mailto:lnwang@sdau.edu.cn%20) ).

**
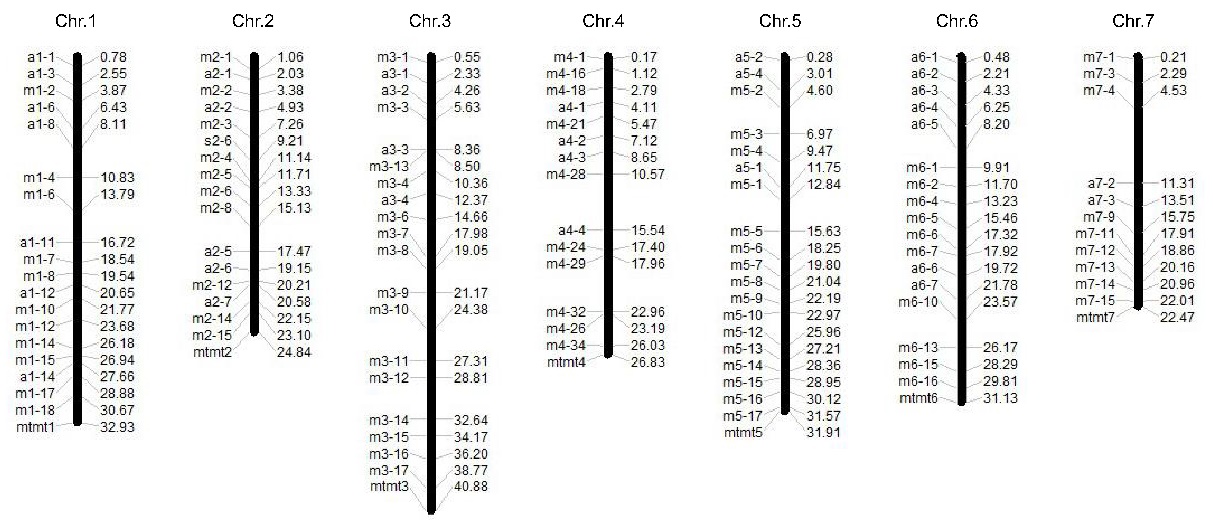
**

**Figure S1. Chromosomal distribution of 114 InDel markers used in this study.** Markers are shown on the left side of each chromosome and their physical locations (Mb) are indicated on the right side based on cucumber 9930 V3.0 draft genome.


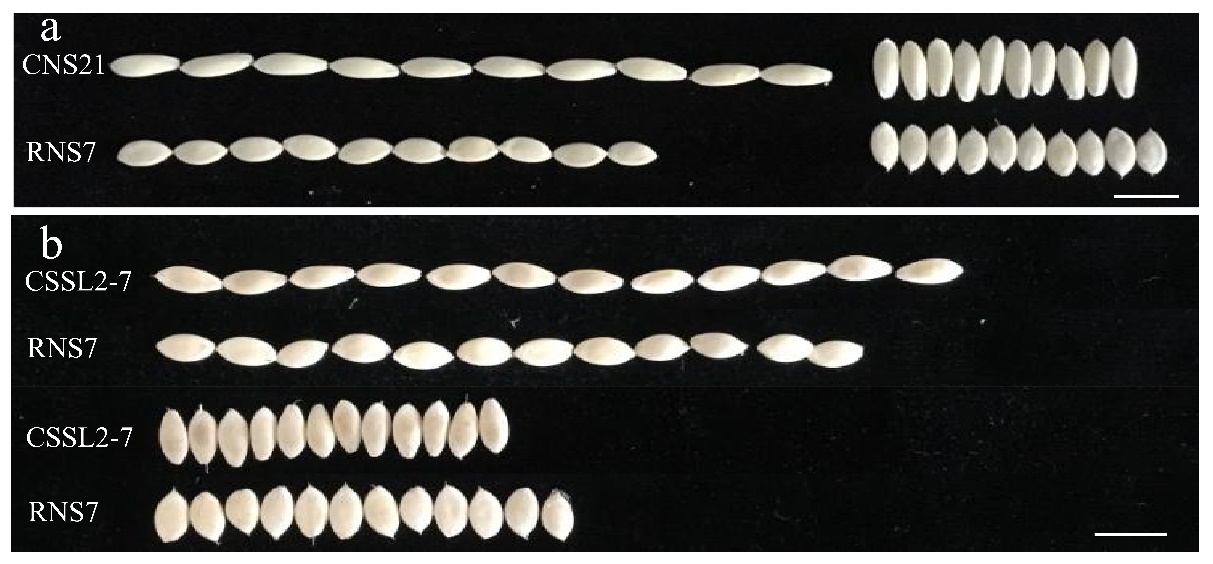


**Figure S2. Seed phenotypes of CNS21, RNS7 and CSSL2-7 carrying QTLs for seed shape. (a)** Seed shape of CNS21 and RNS7. **(b)** Phenotypic comparisons of cucumber seeds between CSSL2-7, a substitution line carrying QTLs for seed shape, and the recurrent parent RNS7. Scale bar = 1 cm.


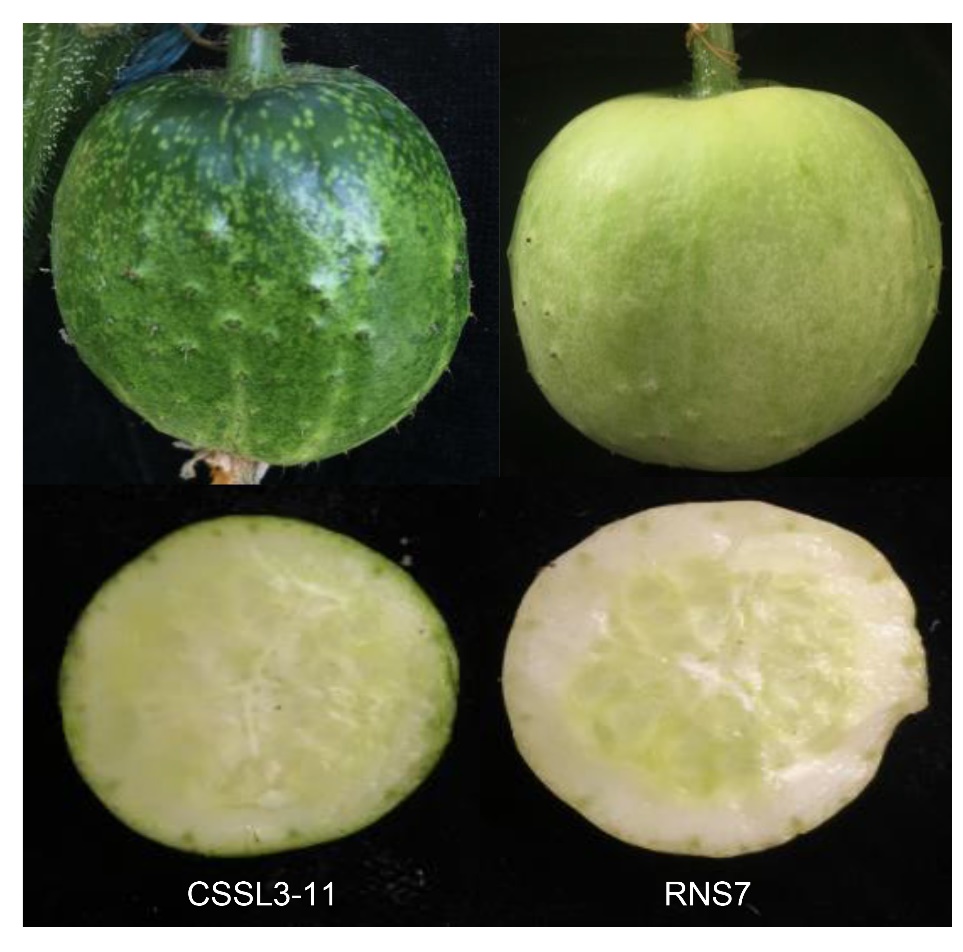


**Figure S3. Comparison of fruit ground and flesh color between CSSL3-11 and the recurrent parent RNS7.** CSSL3-11 is a substitution line that carries QTLs responsible for fruit color.


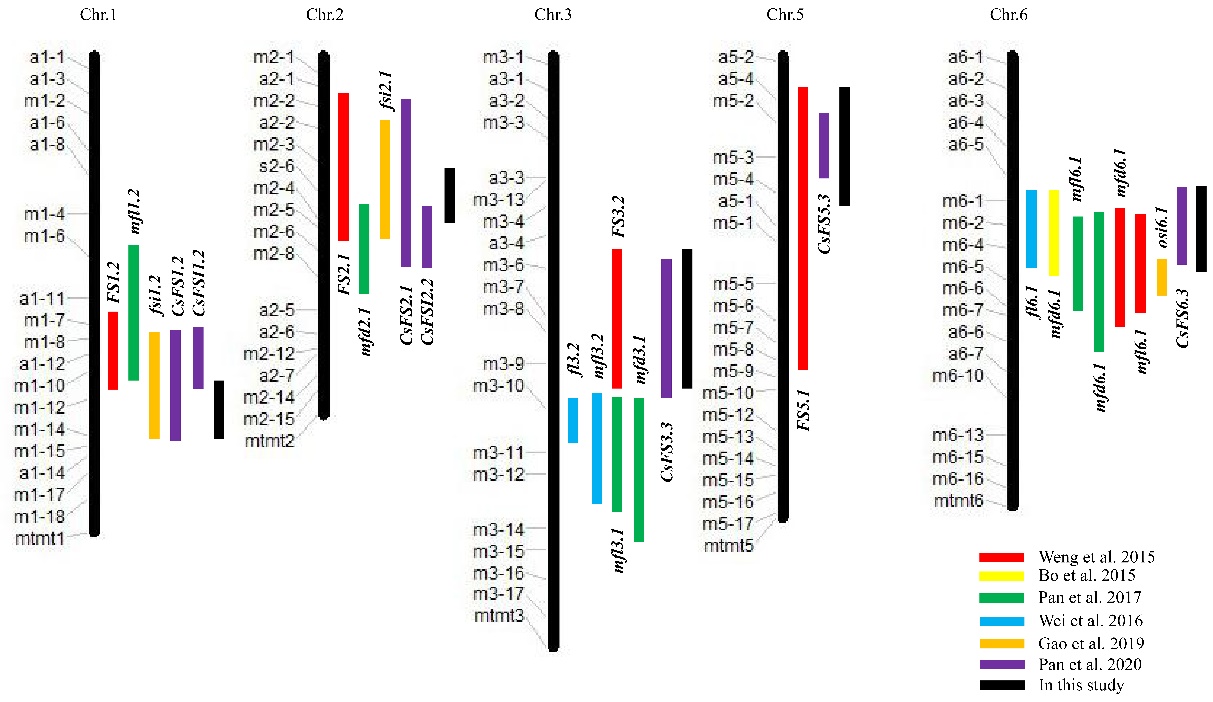


**Figure S4. Chromosomal distribution of cucumber fruit shape related QTLs in previous and the present studies based on cucumber 9930 V3.0 draft genome.**

**Table S1. Distribution of the substituted segments from 71 CSSLs on 7 chromosomes.**

| Chr. | Length of Chr. (Mb) | Number of CSSL | Number of substituted segments | Length of substituted segments (Mb) | | | Times of genome coverage |
| --- | --- | --- | --- | --- | --- | --- | --- |
|  |  |  |  | Rang | Average | Total |  |
| 1  2  3  4  5  6  7  Average  Total | 32.93  24.84  40.88  26.83  31.91  31.13  22.47  -  210.43 | 17  12  11  6  9  7  9  -  71 | 20  12  12  6  10  7  9  10.86  76 | 2.48-19.31  3.29-9.94  4.51-15.85  1.96-18.92  2.36-16.94  3.46-16.39  1.73-14.66  -  - | 7.04  13.52  7.18  7.94  7.98  7.60  7.07  7.19  - | 140.81  75.72  86.10  47.66  79.83  53.17  63.67  78.14  546.96 | 4.28  3.05  2.11  1.78  2.50  1.71  2.83  2.61  - |

Note: Data were analyzed based on cucumber 9930 V3.0 draft genome.

**Table S2. Phenotypic comparisons and additive effects of CSSLs carrying QTLs for cucumber seed shape and fruit color.**

| Line | Chr. | Position (Mb)^a^ | Substituted region | Trait | Add |
| --- | --- | --- | --- | --- | --- |
| **FGC** |  |  |  |  |  |
| CSSL3-11 | 3 | 33.31 ~ 40.88 | m3 - 15 ~ m3 - 17 | Deep green | — |
| RNS7 |  |  |  | Light green |  |
| **FLC** |  |  |  |  |  |
| CSSL3-11 | 3 | 33.31 ~ 40.88 | m3 - 15 ~ m3 - 17 | Light green | — |
| RNS7 |  |  |  | White |  |
| **SDL(mm)** |  |  |  |  |  |
| CSSL2-7 | 2 | 5.10 ~ 14.23 | m2 - 3 ~ m2 - 5 | 9.10±0.10 *** | 0.70 |
| RNS7 |  |  |  | 7.70±0.10 |  |
| **SW(mm)** |  |  |  |  |  |
| CSSL2-7 | 2 | 5.10 ~ 14.23 | m2 - 3 ~ m2 - 5 | 3.70±0.06 *** | -0.40 |
| RNS7 |  |  |  | 4.50±0.04 |  |

Note: ^a^ Based on cucumber 9930 V3.0 draft genome. *** indicate significant differences at the statistical level of 0.001. Traits of interest were described as the means ± standard deviations (n ≥20). Add: additive effect; FGC: fruit ground color (commercial stage); FLC: flesh color (commercial stage); SDL: seed length; SW: seed width.

**Table S3.** **Phenotypic comparisons of 71 CSSLs at anthesis, commercial and mature fruit stages (attached as an .xlsx file).**

**Table S4. The list of InDel markers used in this study.**

| Chr. | Primer Name | Position (9930 V3.0) | Position (Gy14 V2.0) | Forward Primer | Reverse Primer |
| --- | --- | --- | --- | --- | --- |
| 1  1  1  1  1  1 | a1-1  a1-3  m1-2  a1-6  a1-8  m1-4 | 787,992  2,552,949  3,866,546  6,428,077  8,112,320  10,832,509 | 760,273  2,525,812  not available  6,397,845  8,111,410  10,809,897 | CGTAGTCTATCACACAGT  CTCATGTCTGATGAGCTG  ACGTTTCACTGCCTAGAG  GTACACGCCTTTACCAAC  AGTGCAACGCGCTTAATG  TGGTGAATGTCTCACACG | GAGCAATGCTTGCATAGC  GATAGATAACGGCAGTTC  TATTTCCATGAGGTGGCC  TCCAATCCAAGTGTTAGG  TGACCATGTTTGAGTCAC  ATGTTCGACAGTTGGGTC |
| 1 | m1-6 | 13,787,370 | 13,764,428 | ACCCAAGCTAAGTTCTCG | AGTGTCTAGTCGATCCAC |
| 1 | a1-11 | 16,717,840 | 16,914,674 | GATCGCACCTACTATGTC | TTGAACATGTTTCACAACC |
| 1 | m1-7 | 18,541,941 | 18,698,487 | TATGTCGAGTAGCACGAC | ACCTCATCAAGGGTCAAG |
| 1 | m1-8 | 19,543,522 | 19,670,805 | GCACAGGTAGTATCATGG | TTACGCCTTAGTGGTAGG |
| 1 | a1-12 | 20,650,765 | 20,752,599 | ATCCAAGGAAGCTTAACC | ACTAATGATGATACTACC |
| 1 | m1-10 | 21,774,659 | 21,900,360 | TTCTCCTTGCCTTCATCG | ACCGCCAACGTAATGATG |
| 1 | m1-12 | 23,683,554 | 23,728,992 | CCATGAGCCTAATTTGGG | ACGTCTTCACCATCACTC |
| 1 | m1-14 | 26,186,463 | 26,271,572 | CACCACCCATAACTGTTG | CGATAGTTTGGATGTCGG |
| 1 | m1-15 | 26,941,924 | 27,027,053 | TGCTACGCCAGATACTTC | CACCATCACCACAACAAC |
| 1 | a1-14 | 27,660,729 | 27,745,902 | CTTATTCAGTTGCACCTA | GCTGGCAATGGTGGATAC |
| 1 | m1-17 | 28,885,892 | 28,977,020 | GCTAAAATGCCCTCTGTC | GTTTCTCAAGCCAAGCTC |
| 1 | m1-18 | 30,670,452 | 30,804,516 | CATGCTCTACCCCTTTTC | TTAAGTCTCTGGGCTACC |
| 2 | m2-1 | 1,064,446 | 946,610 | GTTGGACCTGAGCATTAC | AGGGTGCAGTCAAAGTTG |
| 2  2 | a2-1  m2-2 | 2,033,538  3,381,449 | 1,914,362  3,180,496 | CATAGCACTATGGCCTTC  ATCATCCATTCCCGTTCC | CTCTAAGACGTGTCTTGC  TCCTGTGATCAACTGTGG |
| 2 | a2-2 | 4,933,235 | 4,748,596 | AGCTTCTAGGACCGTTTG | GGAGTACGAAGTGAGTTA |
| 2  2 | m2-3  s2-6 | 7,267,036  9,213,381 | 7,105,072  9,059,574 | TCAAGAGGGTATTACGGG  GTAGGAGATCACAAGATC | TCATTGCTGAAGTTGGCG  TAGCAGTACTCAACCAAC |
| 2 | m2-4 | 11,148,037 | 11,011,653 | TATTCTCGCTGCTGTCTC | GGTTTATGTTGGCAACGG |
| 2 | m2-5 | 11,717,827 | 11,581,533 | AGGCTGTAGAAGAAGCAG | ACCTCACCTACAAGAGAG |
| 2 | m2-6 | 13,339,578 | 13,187,838 | GTGAACCCAACTCAACAC | ATGCAACTGGGCAAAGTC |
| 2 | m2-8 | 15,138,103 | 15,513,503 | GGCCCAAAAAACAATGGG | ATTCCACCTGTGAGTGTG |
| 2 | a2-5 | 17,474,233 | 27,674,118 | ATGCTTTGGTAGCTCGAG | GAATCGCACGTTTGTGTC |
| 2  2  2 | a2-6  m2-12  a2-7 | 19,156,941  20,213,471  20,585,159 | 29,322,206  30,375,883  30,751,933 | GATCGATAATGCATCATGC  TACACTTTGTTGCCCCTC  CTTGTATCTCCAAGTTAG | TGCTATAATTGTCTGCCTC  AAGAAACGGAGAAGGCAC  GCAATACCTGTATGATGG |
| 2 | m2-14 | 22,152,904 | 32,316,483 | CATTGTCGTCGTTTCCCT | TAAATCATGTCGGTGCGC |
| 2 | m2-15 | 23,109,153 | 33,290,688 | TAAGCACAGTCCTTGGTC | GGCTCTAATGTCTAAGCC |
| 3 | m3-1 | 550,482 | 418,944 | TCTCAGGTGGTTGGATTG | ACACTGATCCCACAGTAC |
| 3 | a3-1 | 2,326,198 | 2,171,256 | ACTCTAAGCTCTTCAAGG | TGGTTACTCAATTTCCTC |
| 3  3  3  3  3 | a3-2  m3-3  a3-3  m3-13  m3-4 | 4,257,380  5,632,177  8,364,472  8,504,467  10,359,735 | 4,116,678  5,482,269  8,220,063  not available  10,200,534 | AGTTAATCGCGTTAATGT  ATGACTGTGCTGGTCTAG  TGAAGTAATTTGCCTCAC  TCATTCAGCGAGGTGATC  GCTGCATTGCTTCAAGTG | GACACCAAGTTTACAACG  CTTGGACATGCAAAGTGG  TCGTAATCAATGTAGCCC  TCCAGGTTGGACAAAAGG  CCCGTTGCTCTTTTTCCT |
| 3 | a3-4 | 12,368,086 | 12,199,074 | AGTCTATCCTTTATCTCTC | CTTAACACAAGATTGAAGG |
| 3 | m3-6 | 14,656,650 | 14,890,893 | TCTCGAAGCATCTCAGAC | TATCCCAAGCCTCAATGG |
| 3 | m3-7 | 17,977,758 | 18,162,337 | TCTGGAAGGAGTTTGAGG | GTCATCGCATATCTGCTG |
| 3 | m3-8 | 19,053,366 | 19,183,699 | AGTGGTTTCCTGTCATGG | CTACTGGCAAAGCAGACA |
| 3 | m3-9 | 21,174,878 | 21,343,460 | AAAAGTAGGAGGTCGTGC | CGCCATCATCTTCAAAGC |
| 3 | m3-10 | 24,383,920 | 24,953,924 | ACACGACAACACTACCTC | ACTCGTAGGTCTTGTTCC |
| 3 | m3-11 | 27,311,809 | 28,144,667 | ACAGGGAGATTTGCCATC | CCGAGCACTAGGAGAAAA |
| 3 | m3-12 | 28,807,425 | 29,651,981 | CATGTGTTTGGCCTTGTG | TGCAGTCATAGGAAGCAG |
| 3 | m3-14 | 32,636,169 | not available | TCGAAGCCTAATTGGTGG | ACCCCAACCTACTCAAGA |
| 3 | m3-15 | 34,172,036 | 35,015,047 | TTGGCCTCGATACCTTTC | TTGGCTCAAGTAGTGTGC |
| 3  3 | m3-16  m3-17 | 36,199,556  38,769,462 | 37,035,816  39,611,675 | GTTGCATAGAGTTGCGAG  ACCGATTCAGGTTGGTTC | ATACGGTGAACGTGGATC  TTAGCTTCCCCAAAGGTG |
| 4  4  4 | m4-1  m4-16  m4-18 | 172,289  1,118,184  2,792,581 | 177,584  1,110,485  2,795,385 | GCGAGGTCATTAACTAGG  TCCAAACCCGTAGATAGG  GACAACTGCAAGGCTTTC | CTCCAACTAGGTTGAAGC  ACATTGGGTAGGGCTTAG  GCATCTAATTTTCCCCCG |
| 4  4 | a4-1  m4-21 | 4,108,202  5,470,148 | 4,230,199  5,599,596 | ATAACCCACGTAGGACAC  ATTCCCAACGTCTTCGTC | TGGATAGTGGTCAGTTTC  GCCTTCATCTTTTCCGAC |
| 4 | a4-2 | 7,120,714 | 7,236,593 | TACACGCATAATCACCAAC | CAAACCATCAATTCATACC |
| 4  4  4  4 | a4-3  m4-28  a4-4  m4-24 | 8,650,484  10,566,428  15,543,861  17,396,821 | 8,887,453  10,923,948  19,907,623  21,735,277 | AGACTTTCAAGGCCTCAC  AAATCGAGAGTTCACCGC  TGGATAGCAAAGACAGCC  CAGCCCTTTCCTATGCTT | GGTTGTTCATATATCACAC  TACTCACACAACTGCTGG  CCTATGTTAACCGAAACC  GAGGTTGCCCTTTGTCAT |
| 4 | m4-29 | 17,963,358 | 22,301,854 | ATCTCAACGCATGGACAG | TGTTCCACATCCATCACC |
| 4 | m4-32 | 22,962,898 | 27,486,259 | TCACCTCCCTTTGATTCC | TGTCTGTGGGTGTATCTG |
| 4 | m4-26 | 23,193,200 | 27,726,972 | GGTTACCTCTGCATGTAG | GAAACTTCCCAAGGTCAG |
| 4 | m4-34 | 26,032,033 | 30,590,901 | AGAATGTGACTAGACCCC | TGGTAGACACACTAGACC |
| 5 | a5-2 | 278,414 | 272,379 | TTCACCAATTGGGCACAC | CAACAAGTAGGGTCCGTC |
| 5  5 | a5-4  m5-2 | 3,005,872  4,595,818 | 3,001,110  4,600,035 | GGATTTCTACTGGAGGAG  TCGACCATAGCACTGTAG | CACCATGTCCTTGGAATG  GTGGAACACCTGTACTTG |
| 5 | m5-3 | 6,974,064 | 6,847,969 | CATCTACCACTCGAATGG | GGGTATGTATGTGAAGCC |
| 5 | m5-4 | 9,472,119 | 8,661,106 | CTGAGTTTGCTTCAGGTC | TGATCTGTAAGCCAGGAG |
| 5  5 | a5-1  m5-1 | 11,747,651  12,840,661 | 11,769,432  12,812,901 | TACTGCATGTAAGGCCTC  TGGGTAGCTGTAACGTCA | ATGGAACGAACACCATAC  TGCGAGAGGAATAAGGGT |
| 5  5 | m5-5  m5-6 | 15,633,612  18,254,002 | 17,775,185  not available | GAGGTTGGTTAAAGGTCC  TGACCAACACGGAAGTTG | GTTGGGGAGACAATCATG  TTTCCTCAGCATCAACGG |
| 5 | m5-7 | 19,804,616 | 21,721,287 | GAGAGGAAGCCTTCTTGT | GGGTTGAACAGTGAGTTC |
| 5 | m5-8 | 21,039,598 | 22,982,502 | GAGCAGCACACAAGAAGT | CCCAAACCTCCACAAGTT |
| 5 | m5-9 | 22,189,721 | 24,127,831 | CATGCGGTGAAGTAGATG | CTCTTTTCTCGATGCCAC |
| 5 | m5-10 | 22,967,265 | 24,907,555 | GCTATACGTTGAGCTTGG | AGTAAGGGAATGTCCCAC |
| 5 | m5-12 | 25,956,763 | 27,914,074 | CCACATTGTAGGGCTTGT | ATAGTCCAAAGGCACACC |
| 5 | m5-13 | 27,205,357 | 29,165,002 | GGGAGACAAAATACCACC | AGCTGTCCTACTCCTTAG |
| 5 | m5-14 | 28,364,958 | 30,187,914 | ATTGGCAAAGGAGGATCG | CACAAACCCAAAAGGCAC |
| 5 | m5-15 | 28,949,697 | 30,771,093 | CAATCTCAAAGGCCAACG | CCACCCAAAATGAGCATG |
| 5  5 | m5-16  m5-17 | 30,122,393  31,568,085 | 31,950,031  33,395,648 | TCCCCACAAGTCAACAAG  CTGCTAATAACGCGCTTC | TCACTTTGCGATGAGAGG  TCATGGTGGAGAAGGTTG |
| 6 | a6-1 | 477,024 | 488,652 | ACCGATCAGTTCAACTCC | ATTCGTCCATCTCCTCTC |
| 6 | a6-2 | 2,213,415 | 2,228,811 | CTGACCAAAGTGTAGCAC | CTGCATTTCCTGACATTC |
| 6 | a6-3 | 4,327,815 | 4,347,905 | TCCACCACACAGACAAAC | CAAAGTCGTATACTCTTG |
| 6 | a6-4 | 6,250,761 | 6,273,139 | CAAGATGCCCACAAATAC | TAAGTGATCTCACAGGAG |
| 6  6  6  6 | a6-5  m6-1  m6-2  m6-4 | 8,201,561  9,906,362  11,700,010  13,231,920 | not available  9,931,326  11,730,089  13,243,288 | TTGGCATCCCGAATAACC  AGACGAACACCCCTAATC  TGGAGGAGATCACCTTTC  GGGTGAATCTGAGCAAGA | GGATTGGGTCTAGGGTTG  ACACTCACACGACTAAGG  GATGATTCTCTTGGGTGC  AACCATCTAAGCCTCTCG |
| 6 | m6-5 | 15,463,166 | 15,680,113 | AAGATGTCAGACGGTGTG | CACATGGTTTGGTGAAGG |
| 6 | m6-6 | 17,320,859 | 18,494,371 | GGAAGTCTTCTGCGTTTG | TGCGTGGTTAGGGTAAAG |
| 6 | m6-7 | 17,919,052 | 19,087,931 | TCTCTTCCAACCAACTCC | GCACTGCACATGATGATC |
| 6 | a6-6 | 19,724,362 | 20,799,657 | GATACATCACAGATAGCTC | GTAAGGACAATTATGGTTC |
| 6  6  6 | a6-7  m6-10  m6-13 | 21,778,854  23,570,622  26,168,106 | 22,865,945  24,658,526  not available | GGACTAACAGTTCTATGC  CATGTGAAGCTCTGCATG  GTGTCCCCTGTTCATCAA | GGCCACAAATCGAGTGAG  GTCGGGAAGAGTAAAGGA  CGGTCTGTGTGACTTCAA |
| 6 | m6-15 | 28,289,042 | 29,582,860 | AGAATCCACATGGCTGTC | ATCGACAAGGTTCGCTTC |
| 6 | m6-16 | 29,812,697 | 31,106,997 | GAGATGCTCAACATCGAG | GTATCGGTGCGAATAAGC |
| 7 | m7-1 | 210,394 | 134,923 | AAGGTTTCACACTCCCTC | TTACTAGGCACCCAAAGC |
| 7 | m7-3 | 2,298,055 | 2,257,411 | TTCATGGAAATCGACCCC | TGGTTCGTTGTCTTGCAG |
| 7 | m7-4 | 4,533,676 | 4,366,933 | AAGAACAGAAACGGGGAG | TTTCCTTTGGGACTGCTG |
| 7 | a7-2 | 11,317,692 | 12,451,415 | CTAGTAACGATGTGATGC | GAAGAGGTGAAGGTGGAG |
| 7  7 | a7-3  m7-9 | 13,516,613  15,754,012 | not available  16,934,458 | CCCTGGCATCAAACTCGT  ATTTTCTGCAAACCCCCG | AGCATCAACAGCTTCCTC  TGAGATGTTTTGGGCCTC |
| 7 | m7-11 | 17,918,470 | 19,114,260 | TTGGAGGAAGGAACTCAC | GACTCCCTTGGTTAAGAG |
| 7 | m7-12 | 18,863,760 | 20,059,820 | ACGTGTTAGTGATGGGCA | AGTTTCTTGCCAGTGCCT |
| 7 | m7-13 | 20,167,561 | 21,377,125 | ATGGGTGATCATCATGGC | TTAGGTAGGAGTGAACCC |
| 7 | m7-14 | 20,968,916 | 22,288,227 | TGGTTGTTTGGGAGATGG | TATGGGTGAATGGGAGAG |
| 7 | m7-15 | 22,016,723 | 23,335,547 | CTCTCTCGTAAGCATTGG | ATTAGGTAGGGTTGGAGG |
